# A Phase II Randomized, Double-Blind, Placebo-Controlled Trial to Evaluate E-Selectin Inhibition with Uproleselan to Reduce Gastrointestinal Toxicity During Autologous Hematopoietic Cell Transplantation for Multiple Myeloma

**DOI:** 10.1016/j.jtct.2025.11.007

**Published:** 2025-11-09

**Authors:** Zachary D. Crees, Keith Stockerl-Goldstein, Meaghan Ryan, Feng Gao, Brandon Christen, Chris Mayeski, Michael J. Slade, Mark A. Schroeder, Ravi Vij, John F. DiPersio, Geoffrey L. Uy

**Affiliations:** Division of Oncology, Washington University School of Medicine in St. Louis, St. Louis, Missouri

**Keywords:** Multiple myeloma, Autologous hematopoietic cell transplant, Melphalan, GI toxicity, Uproleselan, E-selectin inhibition

## RESEARCH COMMUNICATION

High-dose melphalan with autologous hematopoietic cell transplant (AHCT) for multiple myeloma (MM) improves survival but is associated with gastrointestinal (GI) mucosal injury in >90% of patients; increasing treatment-related morbidity and healthcare resource utilization while negatively impacting quality of life (QoL) [1]. Current management strategies focus primarily on palliation. Effective prophylactic strategies are needed.

E-selectin expression is upregulated along the vascular endothelium at sites of acute chemotherapy-associated injury of the GI epithelium, leading to additional cell-mediated inflammatory damage [2]. Uproleselan is a synthetic E-selectin antagonist [2]. Preclinical studies suggest uproleselan, given during chemotherapy, protected mice from weight loss and chemotherapy-induced GI toxicity via inhibition of secondary migration of inflammatory F4/80+ Ly-6C+ macrophages to the GI epithelium [2]. Uproleselan was also evaluated as an adjuvant to 7 + 3 or MEC chemotherapy in acute myeloid leukemia, where only 4/157 (2.5%) patients with acute myeloid leukemia experienced Grade 3 to 4 mucositis relative to historically observed rates of 10% to 30% [3]. Therefore, we hypothesized that prophylactic E-selectin inhibition with uproleselan may mitigate GI toxicity during AHCT in MM.

This study was a phase II, single-center, randomized, double-blind, placebo-controlled clinical trial. Eligible patients were 18 to 75 yr of age with ECOG < 2 and adequate organ function receiving high-dose melphalan and AHCT for MM. Patients were randomized 1:1 to receive either uproleselan + standard of care (SoC) or placebo + SoC. Uproleselan (800 mg) or placebo was administered IV every 12 h starting day −3 through day 0 of AHCT. A single dose of melphalan (200 mg/m^2^) was administered IV on day −2. HSC infusion occurred on day 0. Patients otherwise received uniform SoC management per institutional practice (Supplementary Figure 1). The study protocol was approved by the Institutional Review Board of Washington University in accordance with the Declaration of Helsinki, and informed consent was obtained from all participants. The study is registered at ClinicalTrials.gov (NCT04682405). The full protocol is included in the Supplementary Material.

The primary endpoint of this study was defined as diarrhea severity (CTCAE v5.0, Supplementary Figure 2). Additional endpoints included diarrhea severity by Bristol Stool Scale (BSS, Supplementary Figure 3), patient-reported QoL (GI-specific CTCAE PRO Form v1.0, Supplementary Figure 4), mucositis (CTCAE v5.0), neutrophil engraftment (CIBMTR criteria), hospital length of stay, healthcare resource utilization, *Clostridium difficile* infection, nutritional status, MRD post-AHCT (B-cell receptor sequencing at day +100), 2-yr progression-free survival, 2-yr overall survival and soluble E-selectin levels (ELISA assay). All data were analyzed on the intention-to-treat principle using appropriate statistical tests. Statistical significance was defined as *P* value of <.20 for all analyses, given that one purpose of a pilot randomized phase II trial is to detect signals of efficacy in the experimental regimen for further evaluation [4].

**Figure 1.**
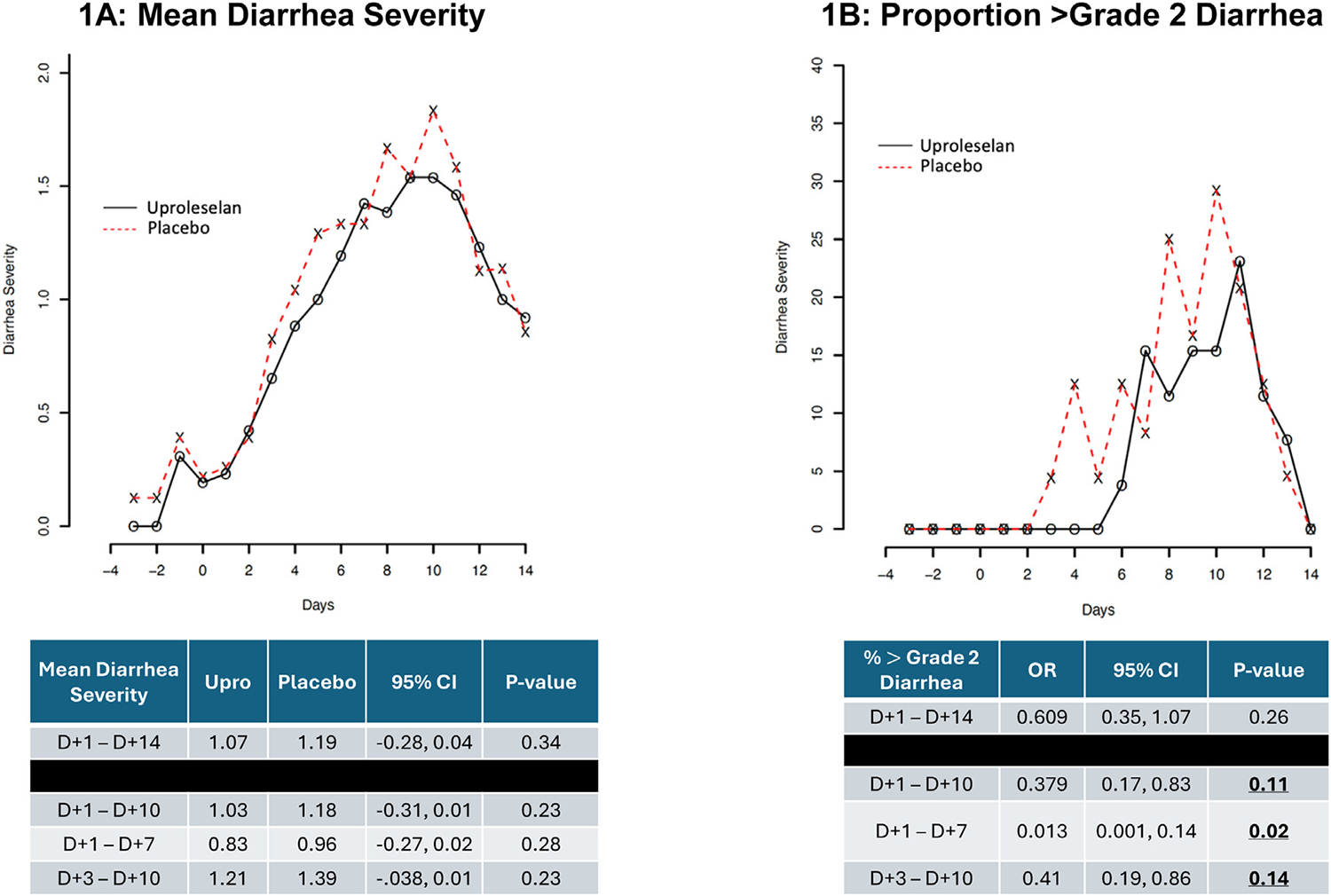
Diarrhea severity was graded via CTCAE v5.0 on a scale ranging from 0 to 5, with Grade 0 = no diarrhea, Grade 1 = <4 stools/day, Grade 2 = 4 to 6 stools/day, Grade 3 = >6 stools/day, Grade 4 = life-threatening, and Grade 5 = death. Part (A) illustrates the mean diarrhea severity score over time in days post-AHCT. Part (B) illustrates the proportion of patients experiencing >Grade 2 diarrhea over time in days post-AHCT. Below the respective figures are data corresponding to the mean diarrhea severity and proportion of >Grade 2 diarrhea, where the day +1 through day +14 timeframe post-AHCT represents the prespecified endpoint. The remaining timeframes below the black bar represent a posthoc sensitivity analysis. Underlined/bolded *P* values indicate meeting the prespecified threshold of *P* < .2.

Fifty patients with MM were enrolled (Supplementary Figure 5). Patient characteristics between cohorts were well-balanced (Supplementary Table 1). The mean diarrhea severity was similar at baseline between cohorts, peaked between days 8 and 11, and improved by day +14 post-AHCT (Figure 1A). The onset of >Grade 2 diarrhea occurred in the placebo cohort on day +3 post-AHCT versus day +6 post-AHCT in the uproleselan cohort, approximately 3 days delayed (Figure 1B). In addition, the proportion of patients experiencing >Grade 2 diarrhea was lower with uproleselan (20.1%) versus placebo (33%). For the primary endpoint, over days 1 to 14 post-AHCT a numerically lower mean diarrhea severity score (uproleselan: 1.07 versus placebo: 1.19 [*P* =.34]) (Figure 1A) and lower incidence of >Grade 2 diarrhea (odds ratio [OR] 0.61, *P* = .26) was observed favoring uproleselan (Figure 1B), but neither met the predefined threshold of *P* < .2. An exploratory sensitivity analysis of diarrhea severity on days 1 to 10, 1 to 7 and 3 to 10 post-AHCT consistently showed numerically lower mean diarrhea severity scores favoring uproleselan (*P* = .23, *P* = .28 and *P* = .23, respectively) (Figure 1A); while >Grade 2 diarrhea incidence was also lower in favor of uproleselan and met the predefined threshold *P* < .2 (*P* = .11, *P* = .02 and *P* = .14, respectively) (Figure 1B).

PRO QoL survey at day +8 post-AHCT showed reduced mean GI-related symptoms in 6/20 (30%) domains (*P* = .07 to 0.14) favoring uproleselan, whereas only 1/20 domains favored placebo (*P* = .17) (Supplementary Tables 2 and 3). Meanwhile, the incidence of severe (Type 7) diarrhea by BSS was reduced with uproleselan (32%) versus placebo (42%) (OR 0.63, *P* = .10), consistent with a shift in the uproleselan cohort toward lower severity diarrhea by BSS (Supplementary Table 4).

Oral mucositis, *C. difficile* infection, time to neutrophil engraftment, and hospital length of stay were similar between cohorts. Time to 1st antibiotic use post-AHCT was delayed with uproleselan (11.5 days) versus placebo (8.0 days) (*P* = .06). Although baseline weight (uproleselan: 88.90 kg versus placebo: 88.00 kg) and overall change in weight were not significantly different (*P* = .60), numeric differences were observed at day +8 (uproleselan: 99.7 kg versus placebo: 79.35 kg) and day +14 or date of discharge (uproleselan: 102.95 kg versus placebo: 81.20 kg) consistent with preserved weight with uproleselan versus weight loss with placebo. TPN was used in 1 patient. MRD negative status at day +100 post-AHCT was similar (uproleselan: 31.25% versus placebo: 26.32%) (*P* = 1.00). The 2-yr progression-free survival was similar (uproleselan: 77% versus placebo: 75%) (*P* = .85). The 2-yr overall survival was similar (uproleselan: 92% versus placebo: 83%) (*P* = .37).

A greater decrease in soluble E-selectin levels occurred with uproleselan (39.9%) versus placebo (20.3%) (*P* < .01), consistent with greater E-selectin blockade in the uproleselan cohort. Notably, greater reductions in soluble E-selectin levels were associated with lower maximum diarrhea severity (*P* = .02), with patients experiencing maximum diarrhea severity score of 1 having median 43.3% reduction in soluble E-selectin levels versus maximum diarrhea severity score of 3 having median 24.7% soluble E-selectin level reduction. Similarly, patients with <20% reduction in soluble E-selectin had greater odds of developing severe (Type 7) diarrhea by BSS versus 20% to 40% soluble E-selectin reduction (OR 2.11, *P* = .02) and >40% soluble E-selectin level reduction (OR 2.65, *P* = .01). In addition, greater reduction in soluble E-selectin levels was associated with a delay in onset of >Grade 2 GI toxicity of any kind, with <20% reduction in soluble E-selectin having median onset of >Grade 2 GI toxicity of 9.5 days versus >40% reduction in soluble E-selectin having median time to onset of >Grade 2 GI toxicity that was not reached (*P* = .01) (Supplementary Figure 6).

Multiple studies have evaluated prophylactic strategies to reduce chemotherapy-associated GI toxicity, often meeting mixed results with some showing early signs of effectiveness that could not be confirmed with larger trials, while others were ineffective or lacked sufficiently compelling data to support their widespread use [5]. There has also been notable heterogeneity in trial design across these studies, making definitive cross-trial comparisons challenging [5]. Cryotherapy, which was SoC in this trial, has been studied with high-dose melphalan and has been reported to reduce oral mucositis severity [5]. Nevertheless, current management strategies largely focus on palliation of GI toxicity once it has occurred, with a continued unmet need for improved prophylactic strategies to reduce melphalan-associated GI toxicity following AHCT for MM.

In this randomized, controlled trial, inhibition of E-selectin with uproleselan + SoC prior to melphalan-conditioned AHCT in MM did not meet its primary endpoint but did demonstrate a trend toward lower GI toxicity in the form of reduced incidence of >Grade 2 diarrhea, reduced diarrhea severity by BSS, and improved GI-specific patient-reported QoL when compared to placebo + SoC. Analysis of soluble E-selectin levels suggests greater E-selectin blockade during the at-risk period, possibly by increasing the duration of uproleselan administration post-AHCT, may enable greater reduction in GI toxicity. Further studies are needed to identify effective prophylactic strategies to reduce GI toxicity for MM patients undergoing AHCT.

## Supplementary Material

1

2

3

4

5

6

7

8

9

10

Supplementary material associated with this article can be found, in the online version, at doi:10.1016/j.jtct.2025.11.007.
