# Supplementary figures and images for: A Phase II Randomized, Double-Blind, Placebo-Controlled Trial to Evaluate E-Selectin Inhibition with Uproleselan to Reduce Gastrointestinal Toxicity During Autologous Hematopoietic Cell Transplantation for Multiple Myeloma
Source: Transplant Cell Ther. Author manuscript; Available in PMC 2026 Apr 21. (PMC13097109; doi:10.1016/j.jtct.2025.11.007)

## Slide 1
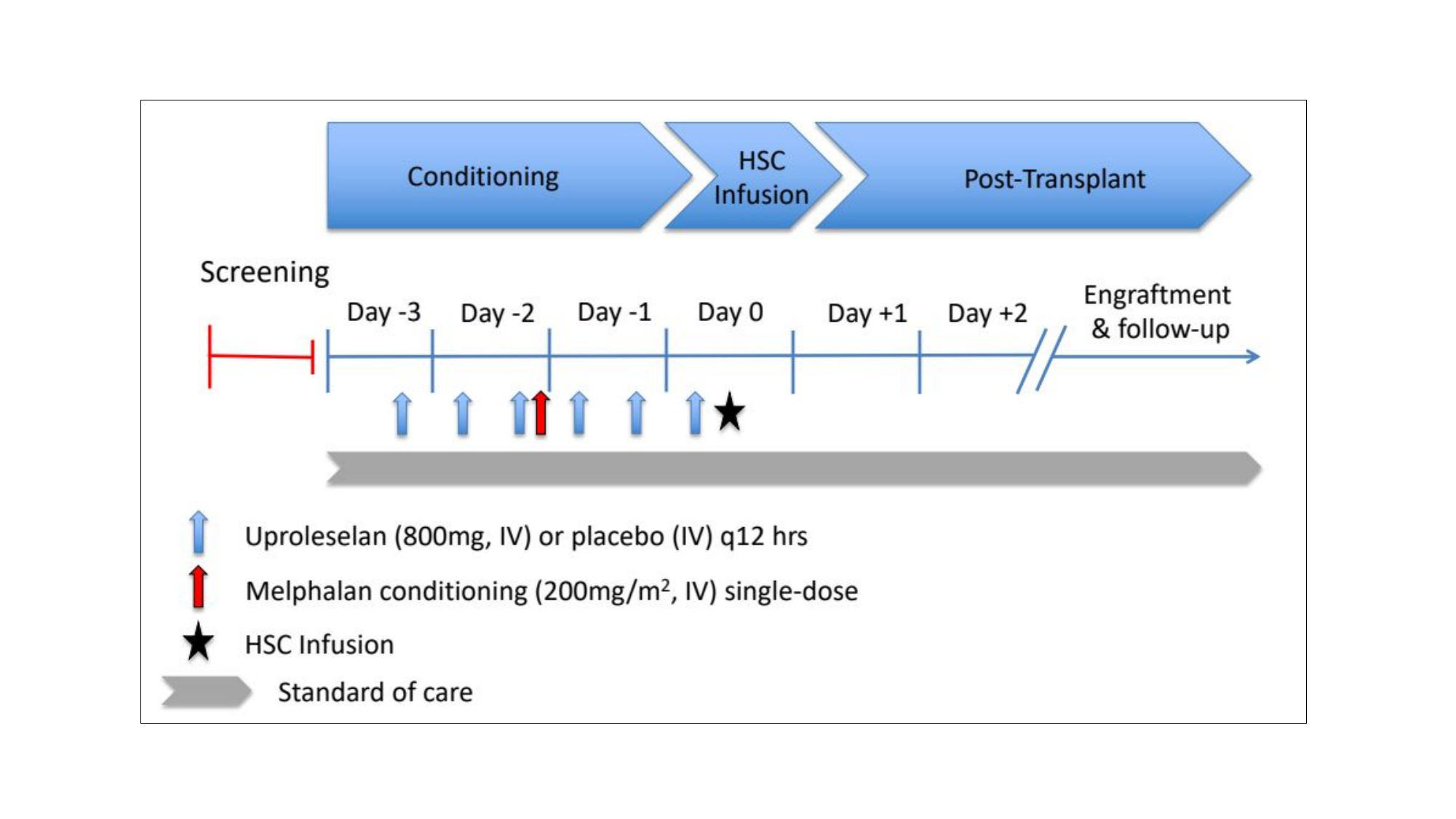

Supplement: 1 [file NIHMS2163084-supplement-1.pptx]

## Slide 1
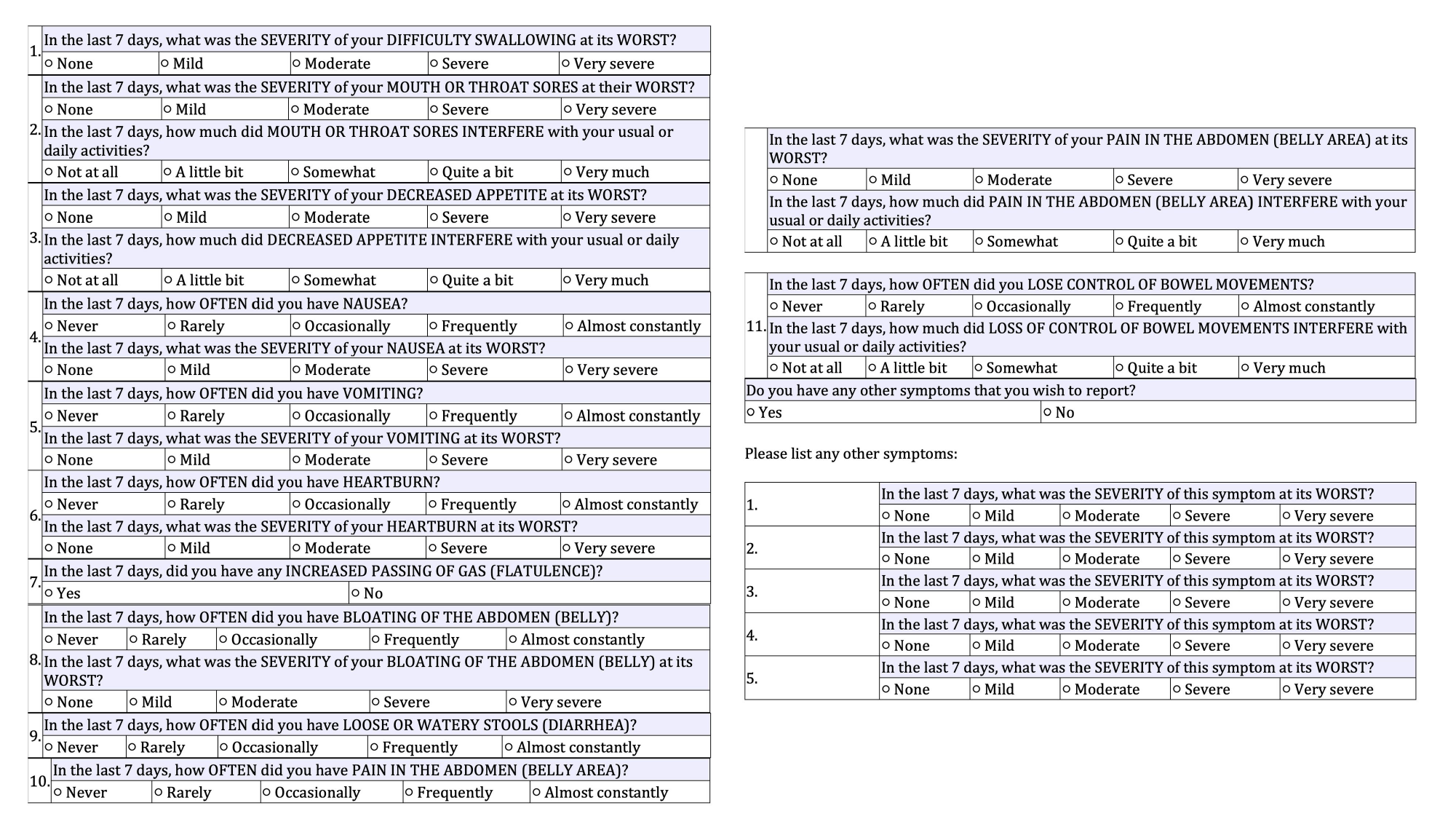

Supplement: 5 [file NIHMS2163084-supplement-5.pptx]

## Slide 1
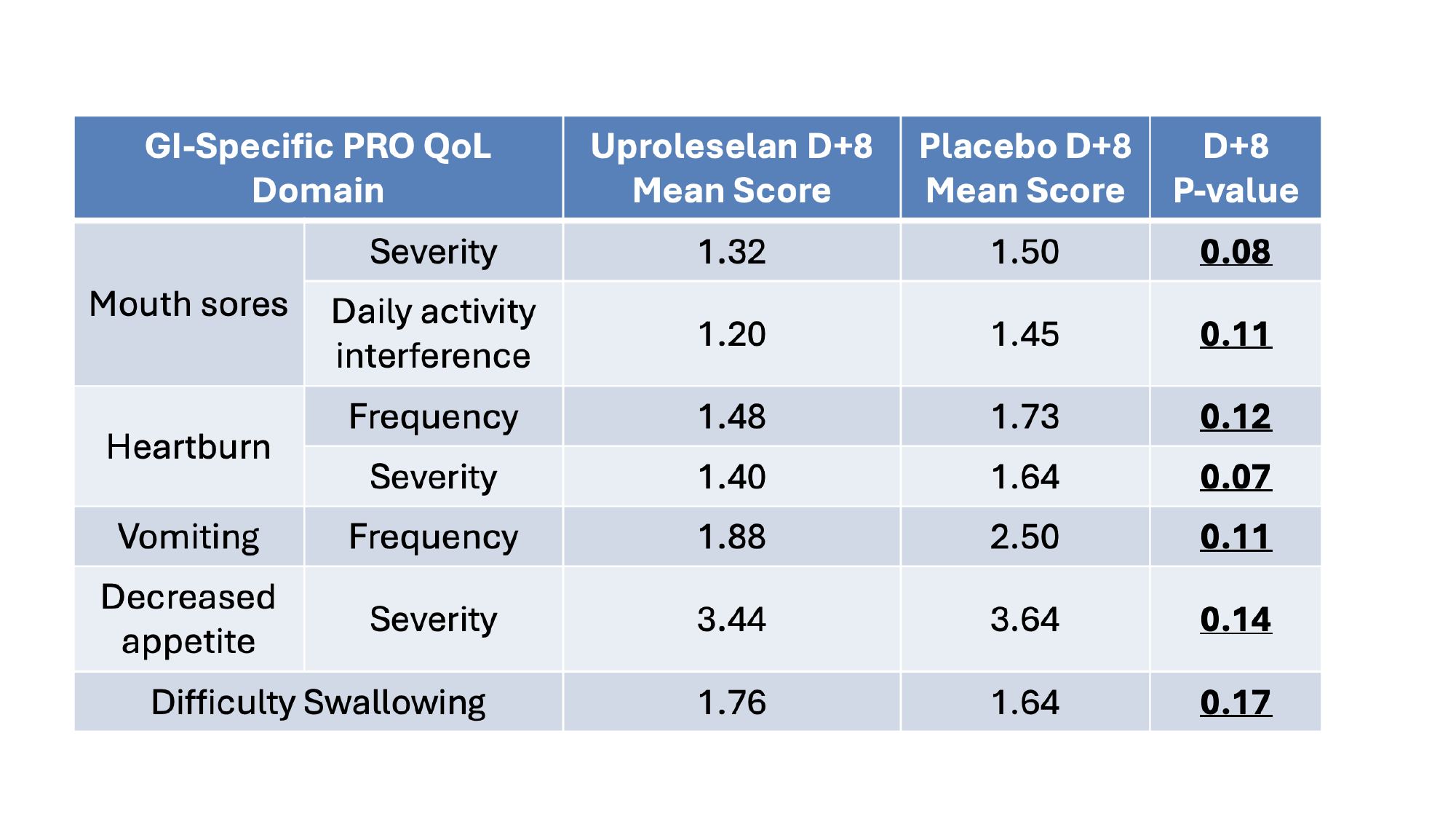

Supplement: 9 [file NIHMS2163084-supplement-9.pptx]

## Slide 1
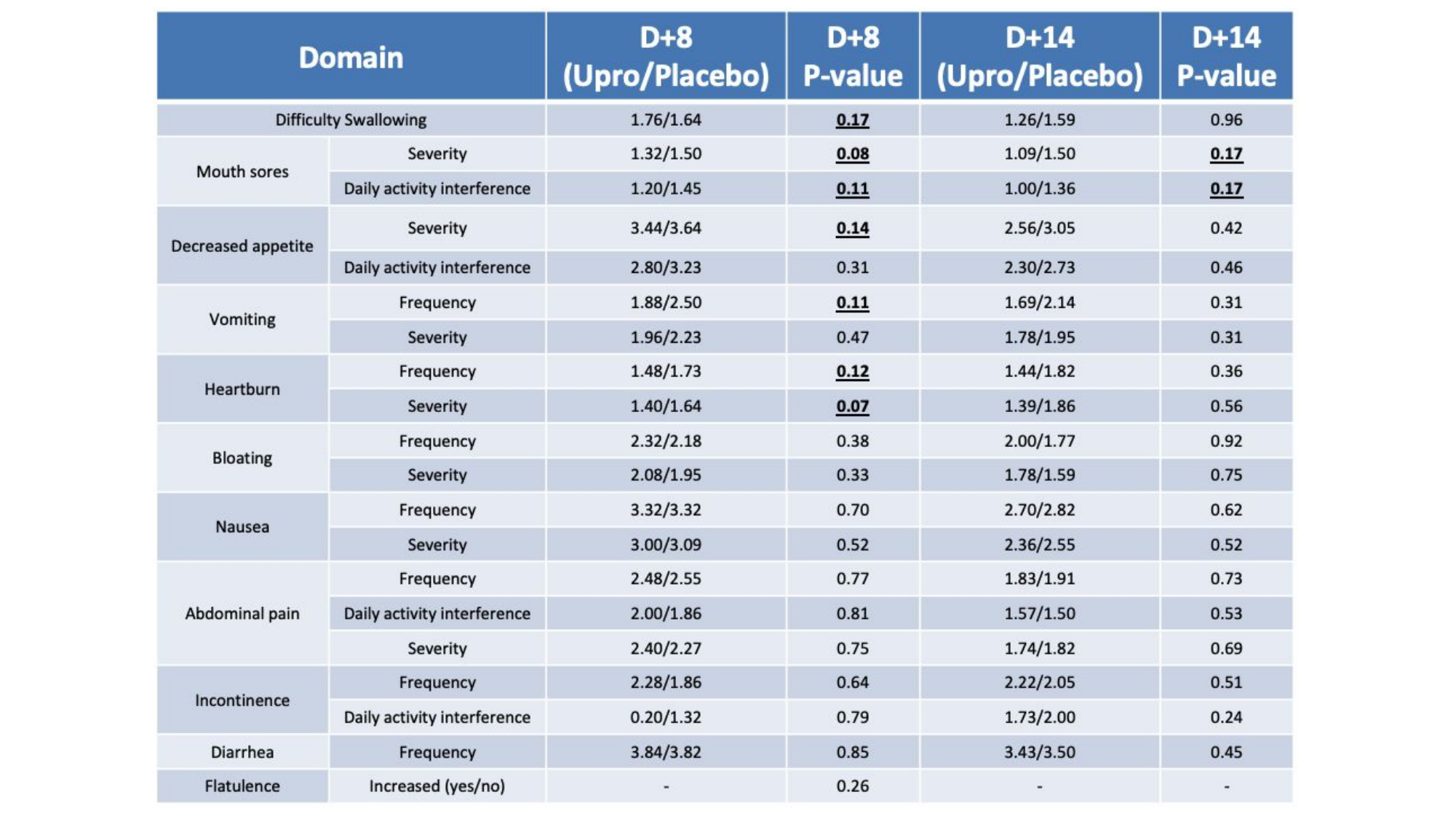

Supplement: 10 [file NIHMS2163084-supplement-10.pptx]
