# Supplementary material for: A Phase II Randomized, Double-Blind, Placebo-Controlled Trial to Evaluate E-Selectin Inhibition with Uproleselan to Reduce Gastrointestinal Toxicity During Autologous Hematopoietic Cell Transplantation for Multiple Myeloma
Source: Transplant Cell Ther. Author manuscript; Available in PMC 2026 Apr 21. (PMC13097109; doi:10.1016/j.jtct.2025.11.007)
Supplement: 2 [file NIHMS2163084-supplement-2.pptx]

## Slide 1
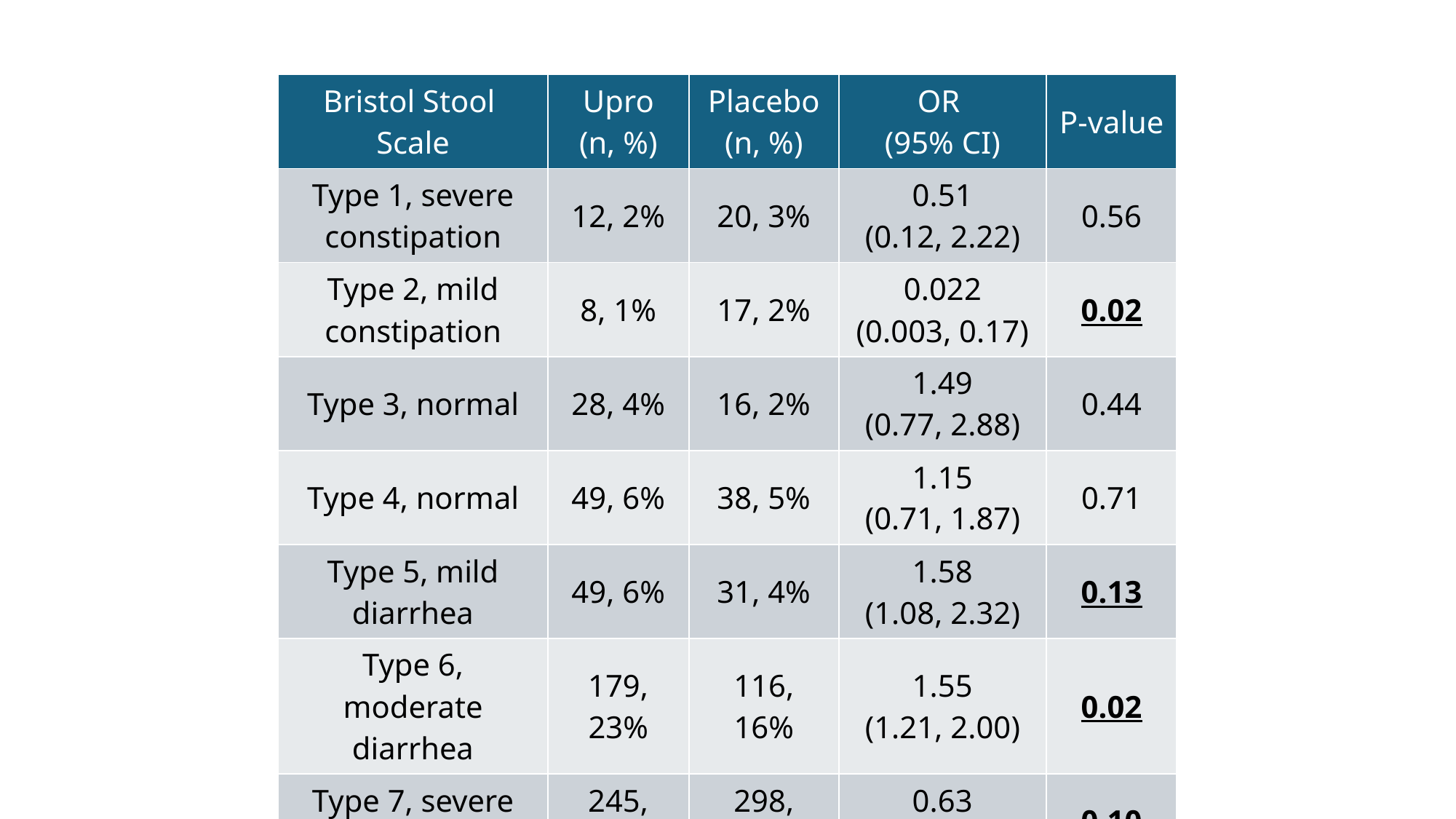

| Bristol Stool Scale | Upro (n, %) | Placebo (n, %) | OR (95% CI) | P-value |
| --- | --- | --- | --- | --- |
| Type 1, severe constipation | 12, 2% | 20, 3% | 0.51 (0.12, 2.22) | 0.56 |
| Type 2, mild constipation | 8, 1% | 17, 2% | 0.022 (0.003, 0.17) | 0.02 |
| Type 3, normal | 28, 4% | 16, 2% | 1.49 (0.77, 2.88) | 0.44 |
| Type 4, normal | 49, 6% | 38, 5% | 1.15 (0.71, 1.87) | 0.71 |
| Type 5, mild diarrhea | 49, 6% | 31, 4% | 1.58 (1.08, 2.32) | 0.13 |
| Type 6, moderate diarrhea | 179, 23% | 116, 16% | 1.55 (1.21, 2.00) | 0.02 |
| Type 7, severe diarrhea | 245, 32% | 298, 42% | 0.63 (0.44, 0.91) | 0.10 |
