# Supplementary material for: A Phase II Randomized, Double-Blind, Placebo-Controlled Trial to Evaluate E-Selectin Inhibition with Uproleselan to Reduce Gastrointestinal Toxicity During Autologous Hematopoietic Cell Transplantation for Multiple Myeloma
Source: Transplant Cell Ther. Author manuscript; Available in PMC 2026 Apr 21. (PMC13097109; doi:10.1016/j.jtct.2025.11.007)
Supplement: 3 [file NIHMS2163084-supplement-3.pptx]

## Slide 1
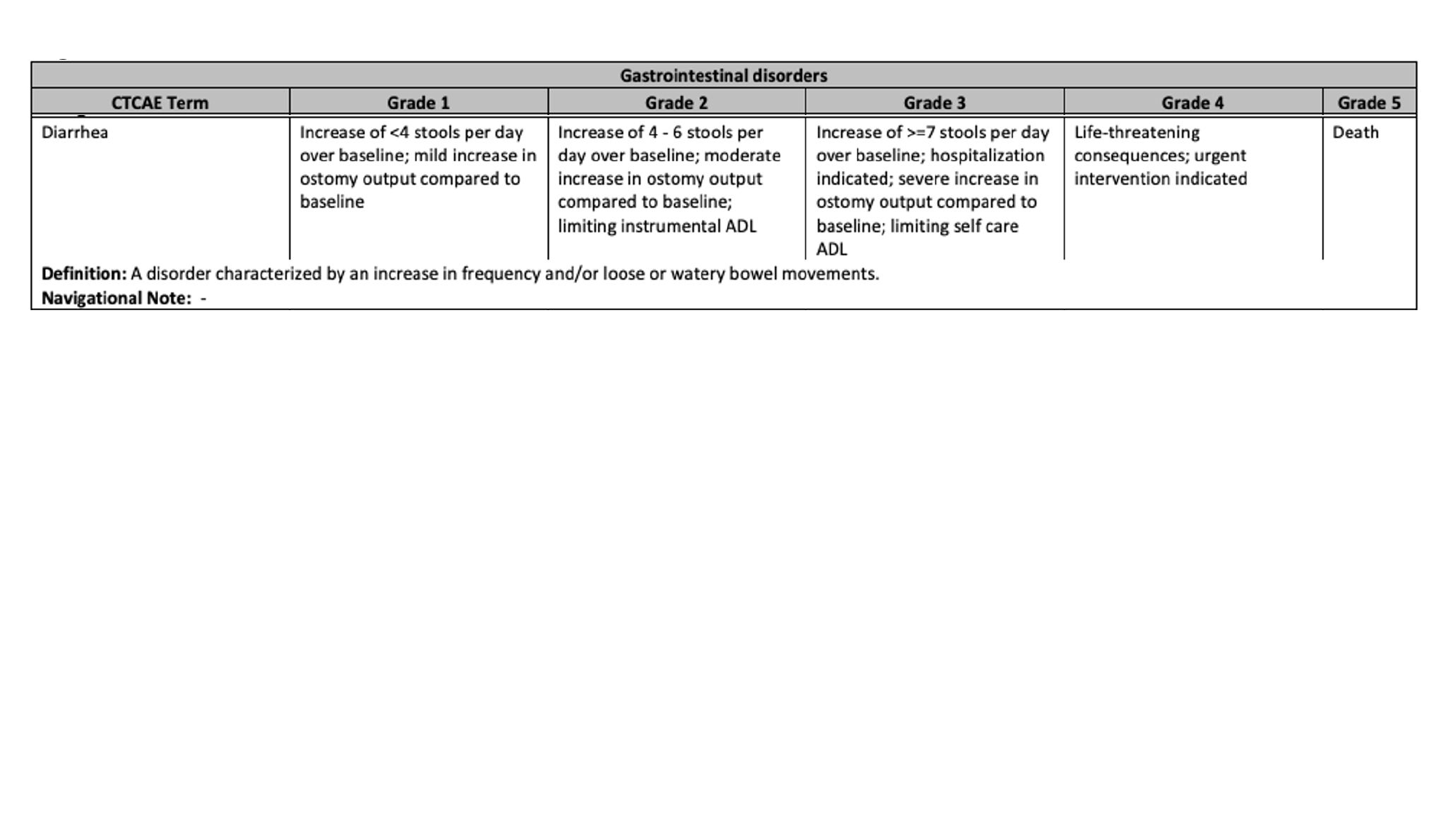

## Slide 2
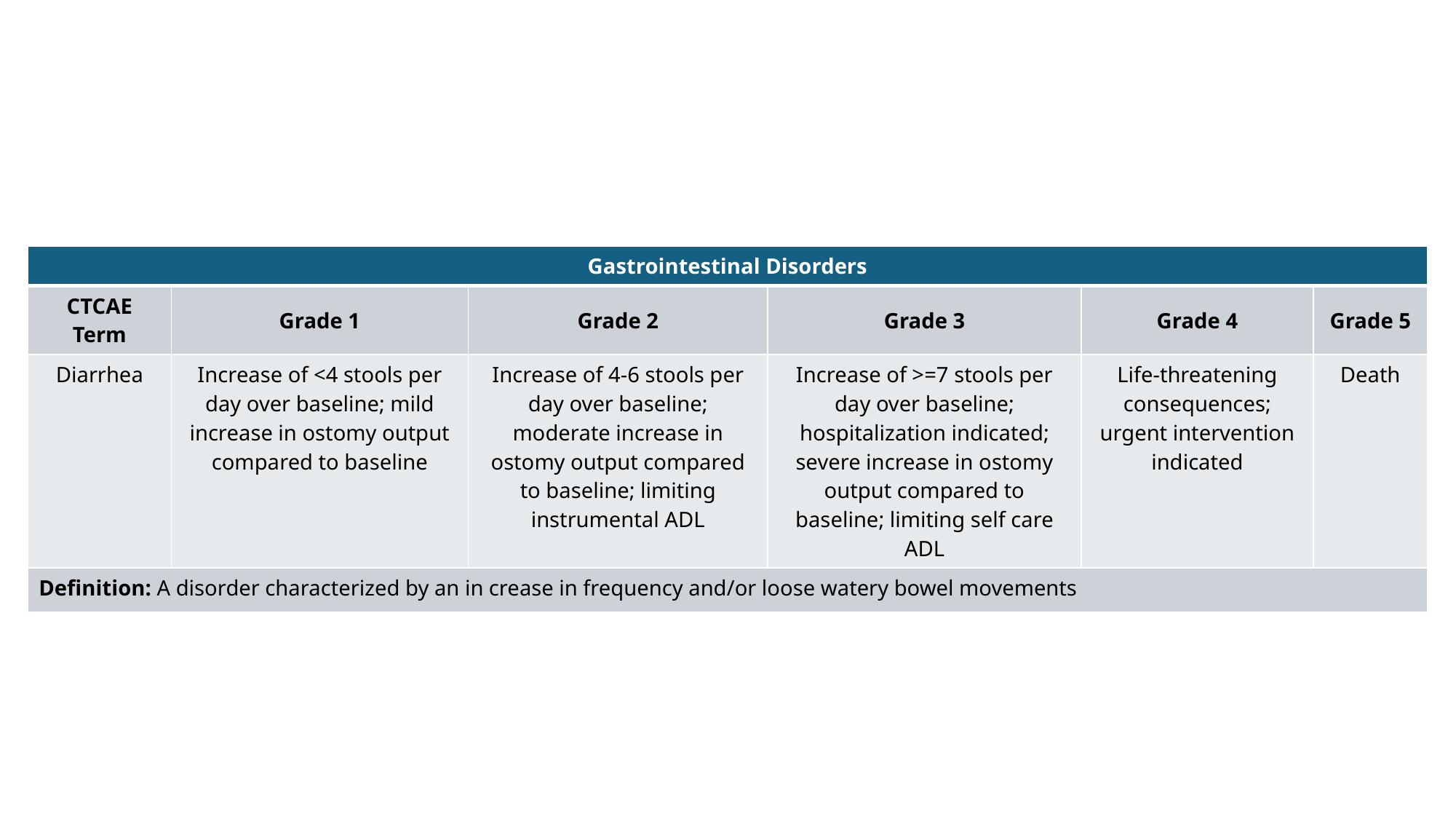

| Gastrointestinal Disorders | | | | | |
| --- | --- | --- | --- | --- | --- |
| CTCAE Term | Grade 1 | Grade 2 | Grade 3 | Grade 4 | Grade 5 |
| Diarrhea | Increase of <4 stools per day over baseline; mild increase in ostomy output compared to baseline | Increase of 4-6 stools per day over baseline; moderate increase in ostomy output compared to baseline; limiting instrumental ADL | Increase of >=7 stools per day over baseline; hospitalization indicated; severe increase in ostomy output compared to baseline; limiting self care ADL | Life-threatening consequences; urgent intervention indicated | Death |
| Definition: A disorder characterized by an in crease in frequency and/or loose watery bowel movements | | | | | |
