# Supplementary material for: A Phase II Randomized, Double-Blind, Placebo-Controlled Trial to Evaluate E-Selectin Inhibition with Uproleselan to Reduce Gastrointestinal Toxicity During Autologous Hematopoietic Cell Transplantation for Multiple Myeloma
Source: Transplant Cell Ther. Author manuscript; Available in PMC 2026 Apr 21. (PMC13097109; doi:10.1016/j.jtct.2025.11.007)
Supplement: 4 [file NIHMS2163084-supplement-4.pptx]

## Slide 1
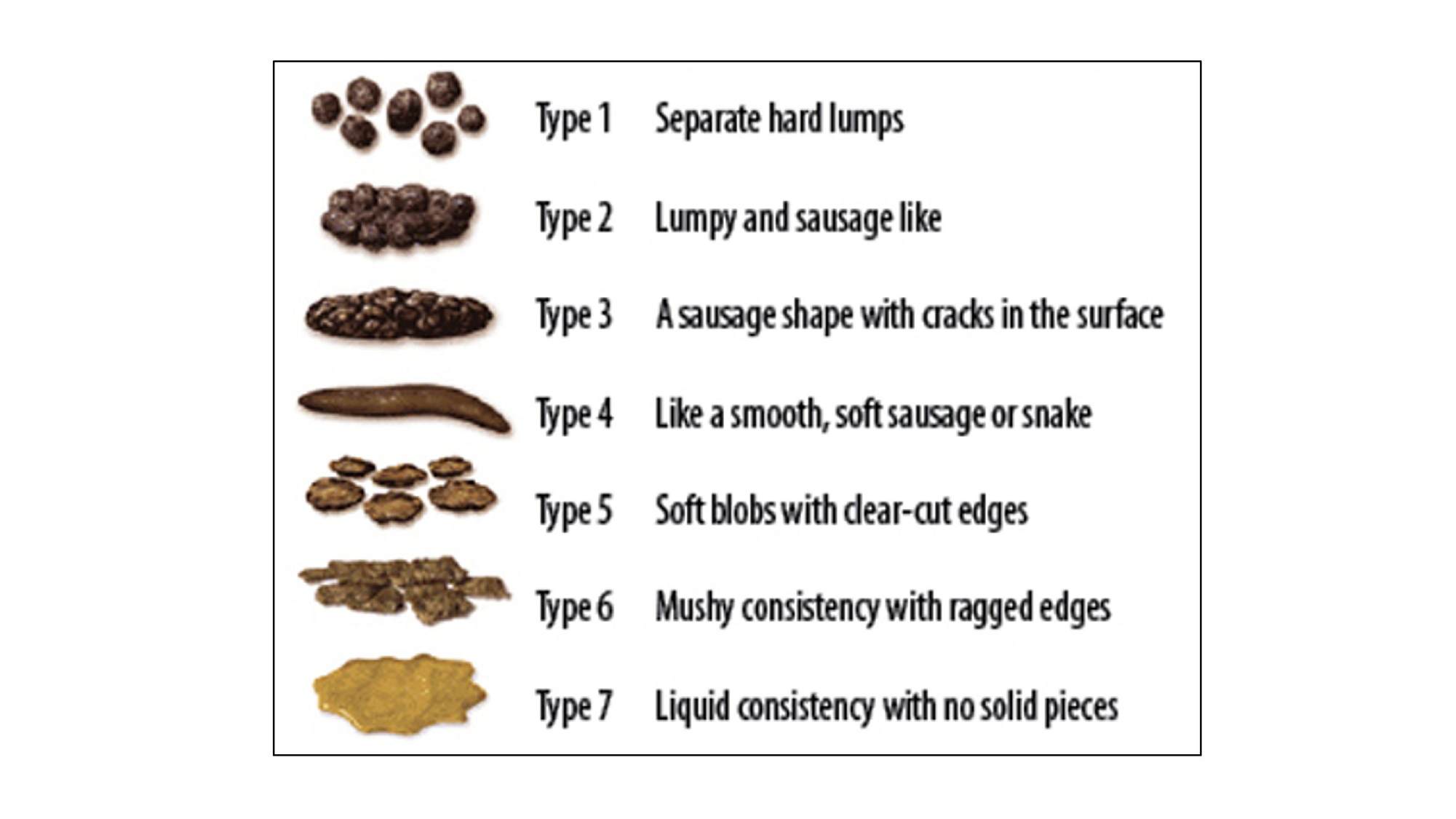

## Slide 2
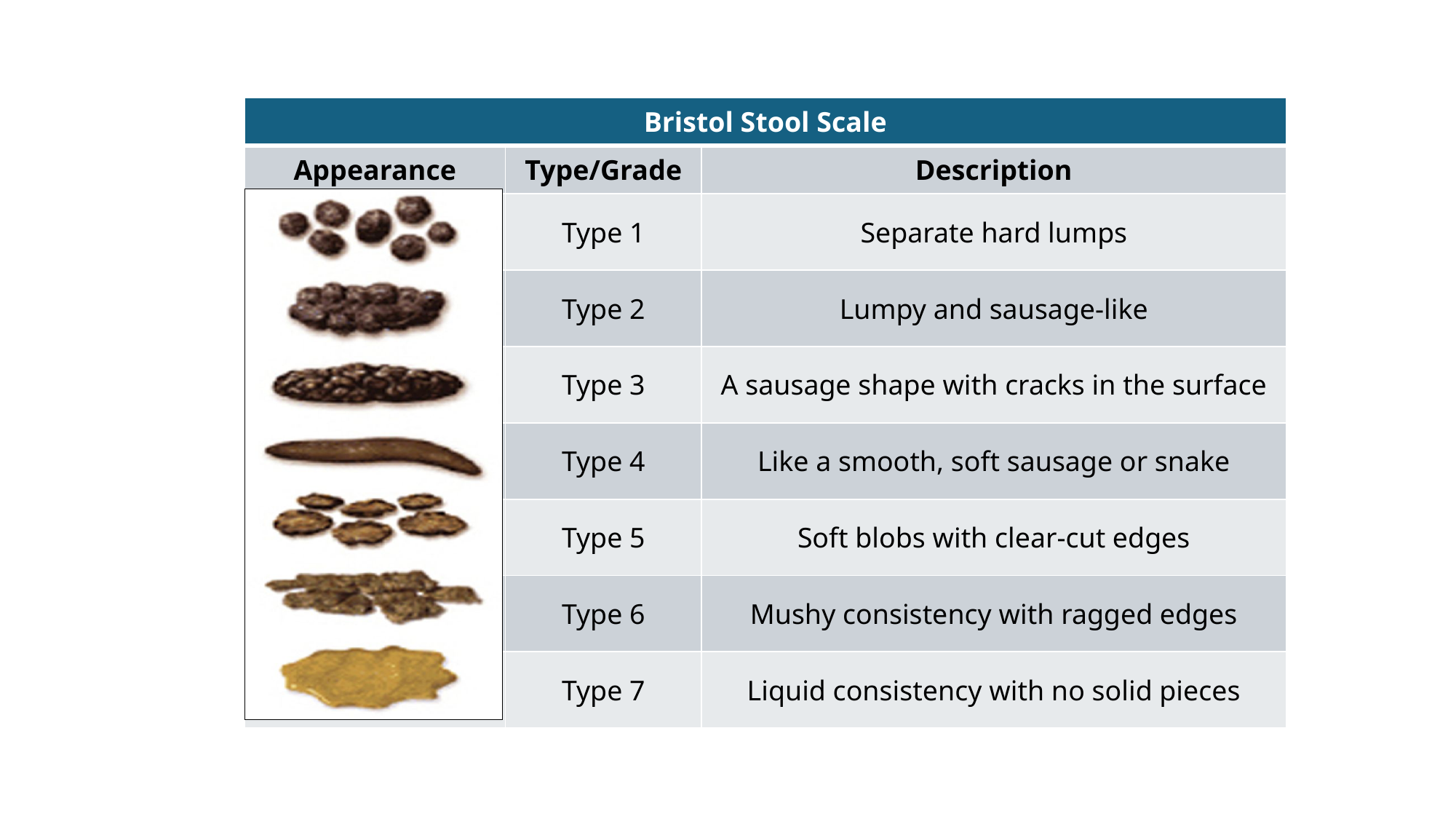

| Bristol Stool Scale | | |
| --- | --- | --- |
| Appearance | Type/Grade | Description |
| | Type 1 | Separate hard lumps |
| | Type 2 | Lumpy and sausage-like |
| | Type 3 | A sausage shape with cracks in the surface |
| | Type 4 | Like a smooth, soft sausage or snake |
| | Type 5 | Soft blobs with clear-cut edges |
| | Type 6 | Mushy consistency with ragged edges |
| | Type 7 | Liquid consistency with no solid pieces |
