# Supplementary material for: A Phase II Randomized, Double-Blind, Placebo-Controlled Trial to Evaluate E-Selectin Inhibition with Uproleselan to Reduce Gastrointestinal Toxicity During Autologous Hematopoietic Cell Transplantation for Multiple Myeloma
Source: Transplant Cell Ther. Author manuscript; Available in PMC 2026 Apr 21. (PMC13097109; doi:10.1016/j.jtct.2025.11.007)
Supplement: 6 [file NIHMS2163084-supplement-6.pptx]

## Slide 1
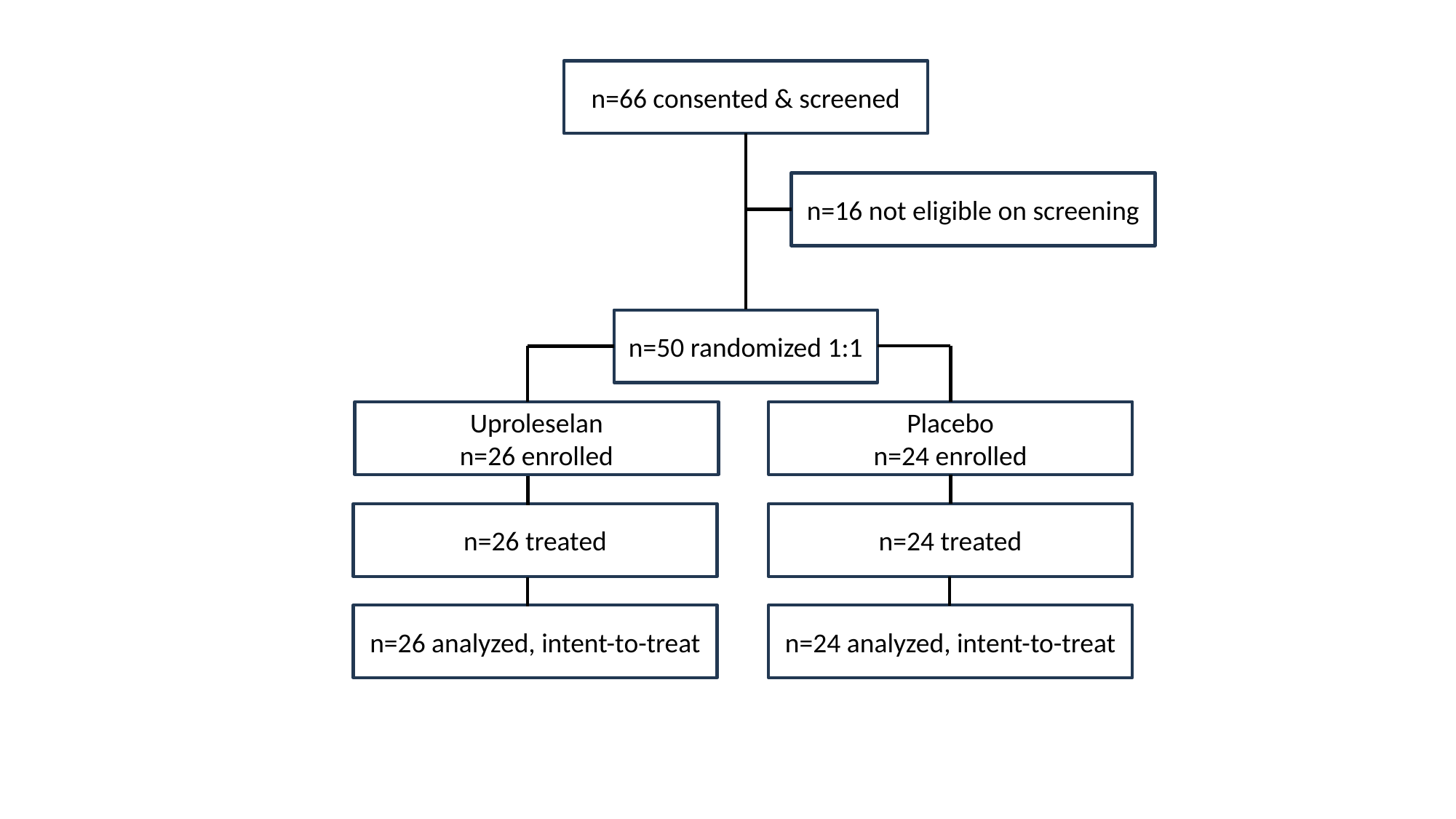

n=66 consented & screened
n=16 not eligible on screening
n=50 randomized 1:1
Uproleselan
n=26 enrolled
Placebo
n=24 enrolled
n=26 treated
n=24 treated
n=26 analyzed, intent-to-treat
n=24 analyzed, intent-to-treat
