# Supplementary material for: A Phase II Randomized, Double-Blind, Placebo-Controlled Trial to Evaluate E-Selectin Inhibition with Uproleselan to Reduce Gastrointestinal Toxicity During Autologous Hematopoietic Cell Transplantation for Multiple Myeloma
Source: Transplant Cell Ther. Author manuscript; Available in PMC 2026 Apr 21. (PMC13097109; doi:10.1016/j.jtct.2025.11.007)
Supplement: 7 [file NIHMS2163084-supplement-7.pptx]

## Slide 1
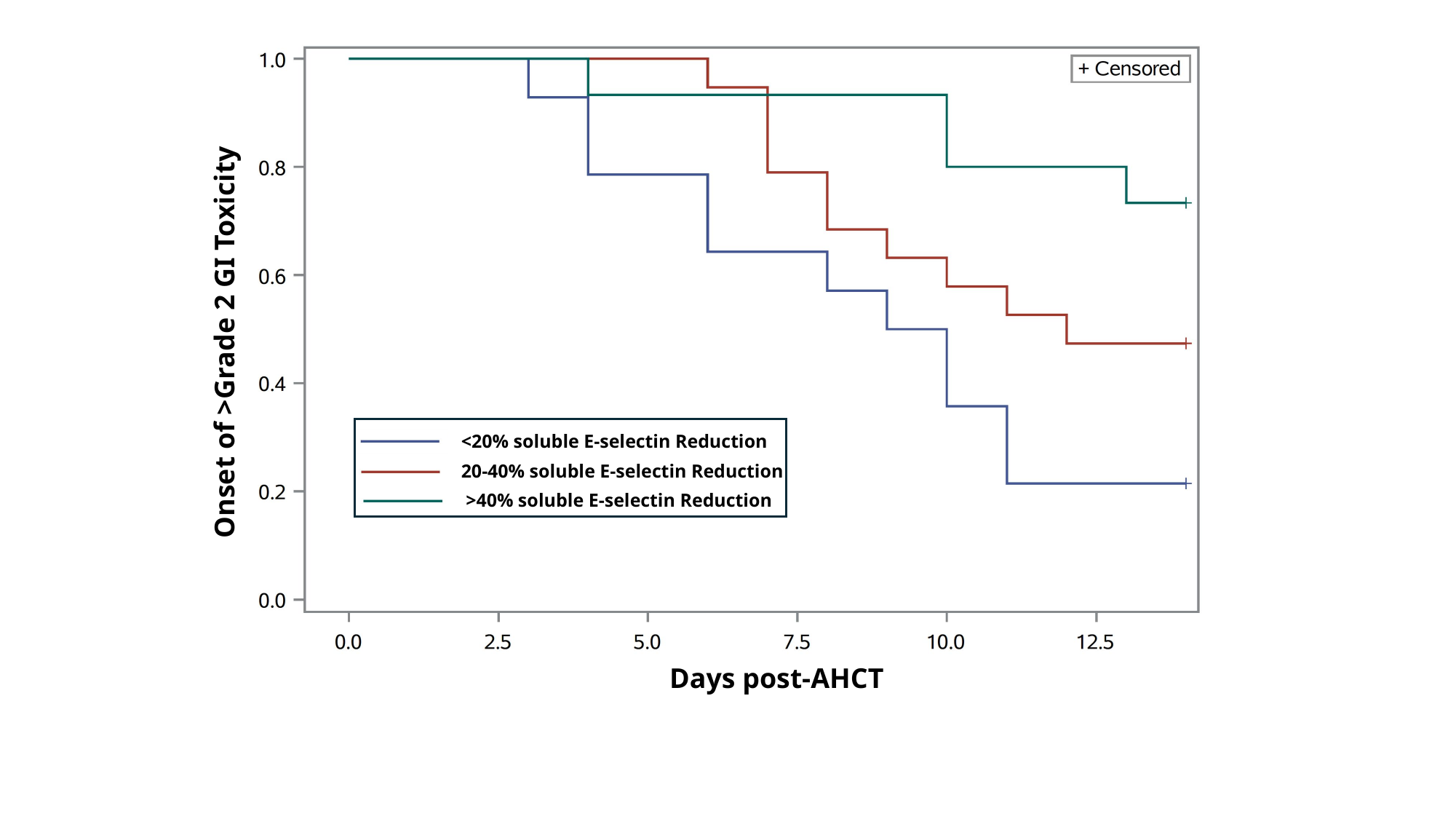

Onset of >Grade 2 GI Toxicity
<20% soluble E-selectin Reduction
20-40% soluble E-selectin Reduction
>40% soluble E-selectin Reduction
Days post-AHCT
