# Supplementary material for: A Phase II Randomized, Double-Blind, Placebo-Controlled Trial to Evaluate E-Selectin Inhibition with Uproleselan to Reduce Gastrointestinal Toxicity During Autologous Hematopoietic Cell Transplantation for Multiple Myeloma
Source: Transplant Cell Ther. Author manuscript; Available in PMC 2026 Apr 21. (PMC13097109; doi:10.1016/j.jtct.2025.11.007)
Supplement: 8 [file NIHMS2163084-supplement-8.pptx]

## Slide 1
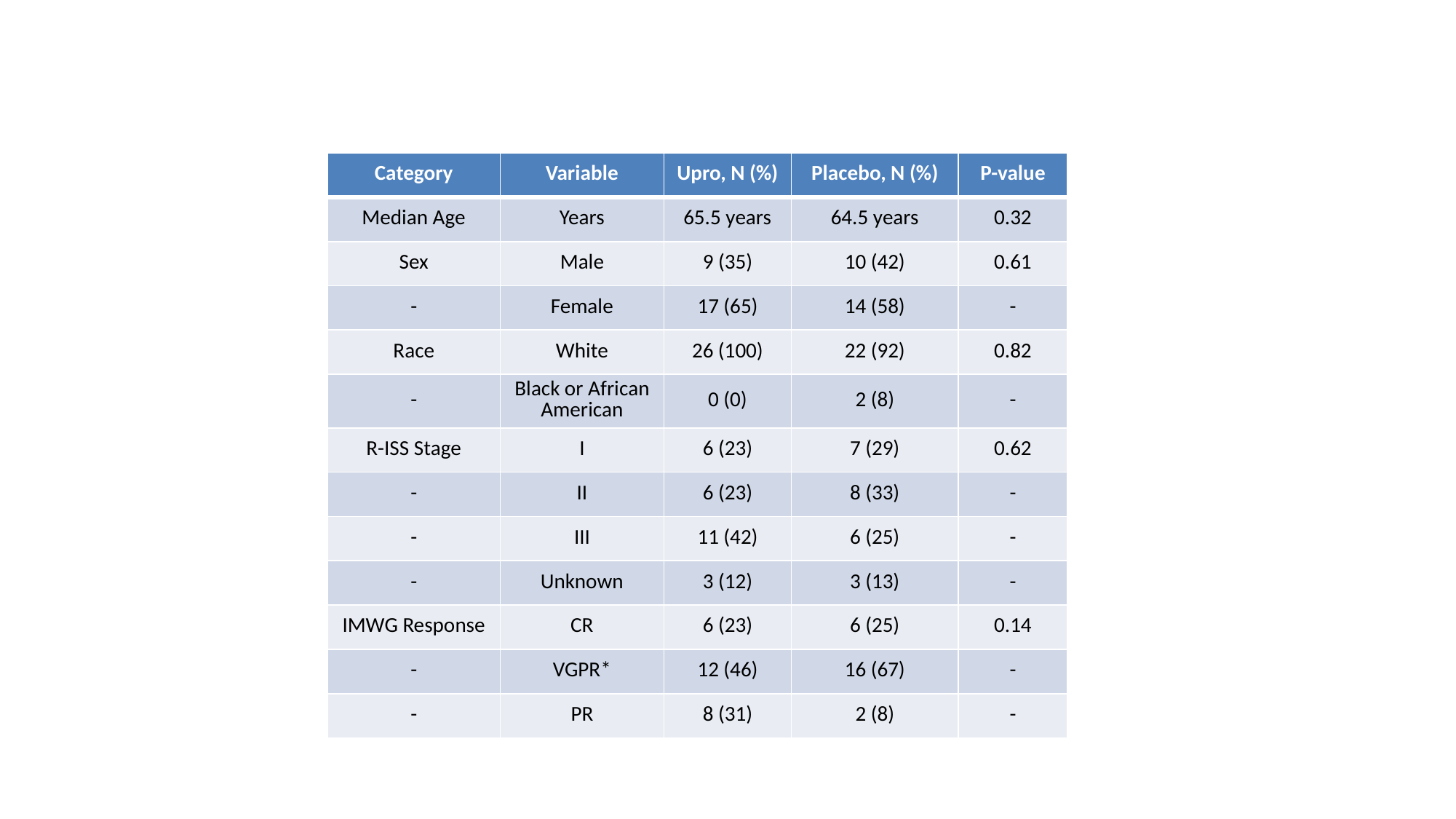

| Category | Variable | Upro, N (%) | Placebo, N (%) | P-value |
| --- | --- | --- | --- | --- |
| Median Age | Years | 65.5 years | 64.5 years | 0.32 |
| Sex | Male | 9 (35) | 10 (42) | 0.61 |
| - | Female | 17 (65) | 14 (58) | - |
| Race | White | 26 (100) | 22 (92) | 0.82 |
| - | Black or African American | 0 (0) | 2 (8) | - |
| R-ISS Stage | I | 6 (23) | 7 (29) | 0.62 |
| - | II | 6 (23) | 8 (33) | - |
| - | III | 11 (42) | 6 (25) | - |
| - | Unknown | 3 (12) | 3 (13) | - |
| IMWG Response | CR | 6 (23) | 6 (25) | 0.14 |
| - | VGPR\* | 12 (46) | 16 (67) | - |
| - | PR | 8 (31) | 2 (8) | - |
